# Effects of the Healthy Start randomized intervention trial on physical activity among normal weight preschool children predisposed to overweight and obesity

**DOI:** 10.1371/journal.pone.0185266

**Published:** 2017-10-09

**Authors:** Mina Nicole Händel, Sofus Christian Larsen, Jeanett Friis Rohde, Maria Stougaard, Nanna Julie Olsen, Berit Lilienthal Heitmann

**Affiliations:** 1 Department of Clinical Research, University of Southern Denmark, Odense Patient Data Explorative Network (OPEN), Odense University Hospital, Odense, Denmark; 2 Research Unit for Dietary Studies, The Parker Institute and the Institute of Preventive Medicine, Bispebjerg and Frederiksberg Hospital, Frederiksberg, Denmark; 3 National Institute of Public Health, University of Southern Denmark, Odense, Denmark; 4 Section for General Practice, Department of Public Health, University of Copenhagen, Copenhagen, Denmark; Weill Cornell Medical College in Qatar, QATAR

## Abstract

**Background:**

There is limited evidence to support the effectiveness of primary interventions aiming to prevent excess weight gain among young children. Evaluating behavioral changes, such as physical activity (PA), may add to future development of efficient interventions. The objective was to evaluate the effect on PA outcomes of the 15 month Healthy Start intervention that focused on changing diet, PA, sleep and stress management among normal weight but obesity-prone preschool children. Children were defined as obesity-prone if they had a birth weight > 4,000 g, mothers with a pre-pregnancy body mass index of > 28 kg/m^2^, or mothers with ≤ 10 years of schooling.

**Method:**

From a baseline study population of 635 normal weight 2–6 year old preschool children from the greater Copenhagen area, parents of 307 children had given information on PA at both the baseline and follow-up examinations. PA was obtained from a 7 days recording in the Children’s Physical Activity Questionnaire. Time used for sport activities were combined with outdoor playing time to achieve a proxy of total PA level of moderate to vigorous intensity.

**Results:**

Linear regression analyses revealed that at follow-up the intervention group spent more time on sports and outdoor activities combined per week than the control group (ITT analyses: intervention: 400 min/week; 95% confidence interval (CI): 341, 459 versus control: 321 min/week; 95% CI: 277, 366; p = 0.02), although no significant intervention effects were seen for each of the subcategories, e.g. sports participation, outdoor activities, screen time, or commuting frequency.

**Conclusion:**

Our results suggest that the overall time spent on sports and outdoor activities combined was increased at follow-up among normal weight obesity-prone children, as a result of the Healthy Start intervention.

**Trial registration:**

ClinicalTrials.gov NCT01583335

## Introduction

According to World Health Organization, there are currently over 42 million overweight preschool children worldwide [1], and developing strategies for the prevention of overweight and obesity should therefore remain a priority. Several systematic reviews and meta-analyses of intervention studies on prevention of overweight and obesity among 0–5 year old children have suggested that the effectiveness of the interventions on primary outcomes, typically body mass index (BMI), seems limited [2–8]. For instance, the meta-analysis from Yavuz and colleagues [3] showed that among mixed weight children at age 0–6 years, and in both short and long-term interventions, the effects were marginal and not of clinical relevance (short-term interventions: Cohens d: 0.08, 95% CI: 0.04, 0.13; long-term interventions: Cohens d: 0.09, 95% CI: 0.02, 0.01). It has been suggested that future primary prevention programmes may be more successful if behavioural change interventions are targeted towards high-risk groups biologically or socially predisposed to obesity [9,10]. Furthermore, even though there may be little or no intervention effects related to change in BMI, the intervention may have induced favorable behavioral changes such as increases in physical activity (PA) or healthy dietary changes [11] that also are considered as important factors in obesity prevention. Indeed, an active lifestyle in children has been linked to other positive health outcomes such as body composition (including smaller waist circumference and lower BMI), cardiovascular indicators and bone health [12–14]. Both favorable and unfavorable PA behaviors, may track from young age into adolescence and adulthood [15–21], thus such early changes can be of potential long-term importance on health. Objective measures, such as those taken by accelerometers, are widely used to assess intensity and time spent being physical active, also among young children. However, apart from the structured activities most likely to be directed by an adult, the characterization of PA among preschool children generally differs from that of older children and adults, by being carried out as mainly unstructured self-selected free playing that can occur at any time or place [22]. Thus, to provide recommendations that can contribute to future development of effective obesity interventions, obtaining information of the habitual PA in regards to types, setting, and frequency may add important information to better understand what specific parts of the activity behavior among young children that are modifiable.

The objective of this study was to evaluate if the Healthy Start randomized primary prevention intervention study improved overall moderate to vigorous activity (proxy) and individual PA categories among 2–6 year old normal weight children, who were all predisposed to later overweight and obesity. Specifically, the outcomes selected were overall activity, sports participation, outside playing time, television and computer use, and commuting choices.

The hypothesis was that during the 15 months of intervention, the intervention group would (a) increase their sports and outdoor activities (proxy variable of moderate to vigorous activity) (b) increase their sports participation, (c) play more outside (min/week), (d) decrease their television and computer use (min/week), and (e) use a more active type of transportation to and from day care (frequency/week) compared to the control group.

## Materials and methods

### Study design

The Healthy Start project was a primary randomized controlled intervention study as it targeted normal weight children, only. The intervention was conducted between May 1^st^ 2009 and August 31^st^ 2011 (participants in the intervention- and control groups were recruited from May to December 2009, and were followed-up from January to August 2011).The overall aim of the intervention was to prevent excessive weight gain among normal weight Danish preschool children who were all predisposed to future overweight and obesity. The Healthy Start intervention project has been described in detail in a previous publication [23]. Briefly, the study was conducted in 11 selected municipalities from the greater Copenhagen area, among children born in the area between January 1^st^ 2004 and December 31^st^ 2007. Through the Danish Medical Birth Registry, all children (n = 22,388) born in the 11 municipalities from 2004 to 2007 were selected. Afterwards, based on the information retrieved from the Danish Medical Birth Registry and administrative birth forms, at least one or more of the following inclusion criteria had to be fulfilled for a child to be invited for enrolment into the study: the child had had a high birth weight (> 4,000 g), and mothers with a pre-pregnancy BMI > 28 kg/m^2^.Furthermore, a subgroup from the municipality of Høje Taastrup was selected based on low maternal educational level (less than 10 years of schooling). Children who had either moved away from the municipality where they were born, had no current permanent address, had died, emigrated, lived at a children's home or were protected from being contacted by researchers were not included in the study. All eligible children living within each municipality strata were randomized (computer-based) into either the intervention or the control group. The children allocated to the intervention group were invited to see a health consultant on a regular basis over a 15 months period with a maximum of 10 visits, and most families received 4 to 5 consultations. The children allocated to the control group met with a health consultant twice; at the beginning of the study and at follow-up on average 15 months after their first visit.

The content of the consultations in the intervention were based around four main themes: optimizing diet and PA, in accordance with the official national recommendations, together with sleep and stress management. For PA among children aged 5 years or older, the official national recommendation is a minimum of 60 minutes activity of moderate to vigorous intensity [24]. The intervention was a non-standardized package and focused on the entire participating family’s individual needs and resources, and hence not only on the child. The theoretical foundation for the health behavior change in the intervention was *Stages of Change* as well as *Motivational Interviewing* [25] to improve the family´s knowledge and action. Each of the four consultation themes had selected key points. The most relevant key points related to this study were to: increase the time the child spend outdoor, reduce the child’s television viewing and promote an active type of transport (walking or biking) [23]. The families in the intervention group were also invited to participate in optional monthly play events as a supplement to the consultations. The purpose of the play events was to convert theory obtained from the consultations into practice and to encourage the families to be more physically active together. Examples of different games that could be played at home were tennis with a balloon, dance freeze, and imitate an animal, e.g. jump like a frog (www.sundstart.nu).

**Fig 1:**
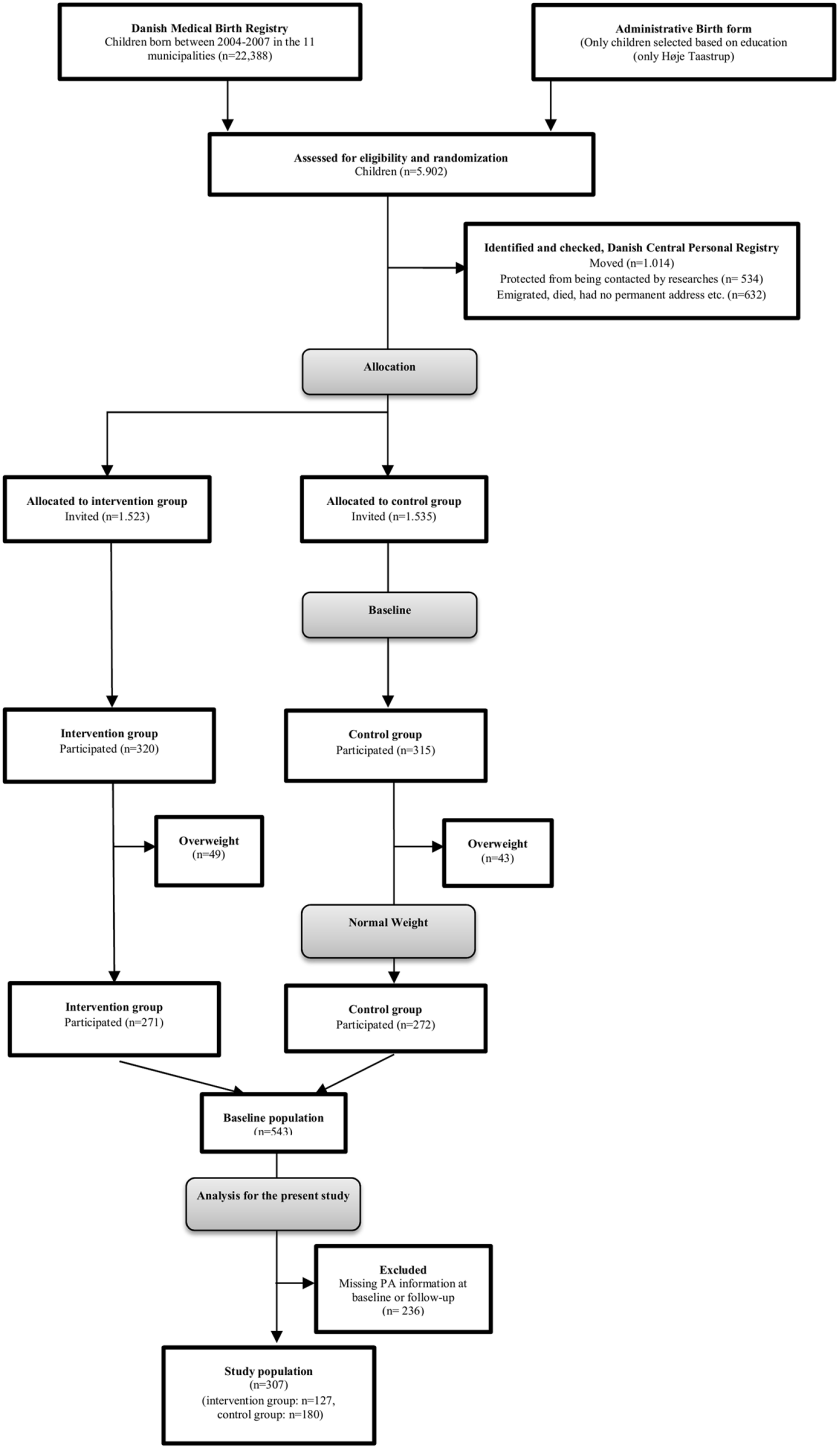
Flow chart of the study population.

Invitations to participate were send out to 3,058 individuals, and in total 635 families accepted the invitation to participate. No incentives were offered for participation. Because the intervention targeted the whole family, siblings to the included children were allocated to the same group (n = 15). Children who at their first meeting with the health consultant (baseline) were found to be overweight according to cutoff values for BMI proposed by Cole et al. [26], were excluded from the current analyses (n = 92), as all children enrolled had to be normal weight at baseline. Moreover, children with missing information on one or more of the PA outcomes at baseline and follow-up were excluded in the per protocol (PP) analyses (n = 236), leaving a study population of 307 individuals with complete information on PA at both the baseline and the follow-up examinations (intervention group: n = 127, control group: n = 180) (flowchart presented in Fig 1).

### Physical activity assessment

At baseline and follow-up, a parental questionnaire holding information on several individual and family related topics was completed.

To obtain information about the PA categories, the Children’s Physical Activity Questionnaire (C-PAQ), consisting of a 7-day recording of sports, games and leisure time activities in the time outside of day care [27], was used. Information on day care participation or time spent in daycare was not obtained. However, a report from SFI—The Danish National Centre for Social Research showed that in 2012 almost all Danish children at age 2 to 6 years were enrolled in a daycare (>93%) [28]. The C-PAQ questionnaire was revised to match the possibilities and types of active and inactive activities, characterizing Danish preschool children.

The response options were indicated by doing a particular activity (“yes” or “no”) and by an indication of total time used on the activity during the week (minutes per week). If the time used on the activity was reported as a time interval, the mean value was chosen. Information on the type of transportation used to bring the child to and from day care was also obtained from the C-PAQ.

#### Outcome variables

The variables described below were generated in order to examine the effectiveness of the intervention on the PA categories among the children (overview is given in Table 1).

**Table 1:** Content of the generated variables sport activities, outdoor playing activities, television and computer use, active and passive transport.

Variable	Included the following activities
Sport activities, min/week^a^	Handball, Dance, Football, Gymnastics, Martial art, Swimming, Horseback Riding, Music and Movement & Ice Skating
Outdoor playing activities, min/week^a^	Bicycling, Trampoline, Playhouse, Playground, Rollerblade, Jump Rope, Hide and Seek, Walk with the dog, Go for a walk
Television and computer use, min/week^a^	Playing computer games, watching television
Active transport, frequency/week^b^	Walking or bicycling
Passive transport, frequency/week^b^	By stroller, bicycle, bus, car or train

^a^ weekdays and weekends combined

^b^ weekdays only

From C-PAQ, information on the child’s weekly participation in sports activities in the child’s leisure time was obtained. The variable “sport activity” was generated by merging all the information obtained about the child’s leisure time participation in sports activities during the week, measured as minutes per week (weekdays and weekend days combined) spent on sports activities. The variable “outdoor playing activities” was generated in the same way as the variable sports activity, and measured the minutes per week (weekdays and weekend days combined) the child spent on the different leisure activities outdoors. Sports activities and outdoor playing time were combined to generate a proxy of total PA level (PAL) of moderate to vigorous intensity. The variable “television and computer use” was generated to estimate time spent on sedentary activities. The variable indicates the minutes per week (weekdays and weekend days combined) the child used on computer games and/or watching television and/or video. Transport of the child to and from day care was categorised in to being either active or passive transport and measured as the frequency to and from day care per week (weekdays only, range 0–10 times). The “active transport” category included transport where the child was physically active, i.e. walking or biking. In contrast, the “passive transport” category included transport where the parents were transporting the child, such as by car, public transportation or on the parents’ bicycle.

#### Covariates

Information on age, gender and municipality of residence for the participating children was retrieved from the Danish Medical Birth Registry.

Information on maternal PA at baseline was obtained from the parental questionnaire, where the parents were asked how to describe their level of PA in leisure time, seen over the past year, based on the following four response options: 1) Train hard and participate in competitive sports regular and several times a week, 2) Perform exercise sports, bike to work or do heavy gardening or similar, 3) Do walks or other light exercise at least 4 hours per week (including Sunday walks, easier gardening or walk to work), or 4) Have other sedentary activities (e.g. reading, watching TV). The four response options were afterwards dichotomized. Response options 1 and 2 were collapsed into “physically active”. Response options 3 and 4 were collapsed into “sedentary”.

Information on maternal education at baseline was obtained from the parental questionnaire, and grouped into “low” and “high” educational level, and cut-off was lower than 10 years, equivalent to secondary school or E-level.

Maternal BMI was calculated using self-reported weight and height obtained from the parental questionnaire at baseline and when not given the pre-pregnancy value from the Medical Birth Registry was used.

Information on distance between home and day care was obtained from the parental questionnaire where the response options were *1) 0–1 km 2) 1–2 km 3) 2–3 km 4) 3–4 km 5) above 4 km*.

Height and weight of the child were measured during the health examinations. Height was measured to the nearest 0.1 cm by using a stature meter (Soehnle 5,002 or Charter ch200P). Weight was measured to the nearest 0.1 kg by using a mechanical weight or a beam scale (TanitaBWB-800 or SV-SECA 710). The child was measured in bare feet or stockings, and children who wore nappies were changed just before weighing. BMI z-scores were generated to provide a relative measure of adiposity for age and time between baseline.

### Ethics

The study complied with the Helsinki II declaration and was approved by the Danish Protection Agency (present: no. 2015-41-3937; previous: no: 2007-41-0530). According to Section 2.-(1) of the Danish Act on a Bioethics Committee System and the Processing of Bioethics Projects, the project was defined not to be a bioethics project and as a result did not need approval from the Scientific Ethical Committee for the Capital Region of Denmark (May 14^th^ 2007, Journal no.: H-A-2007-0019). Study protocol has been published [23]. All parents of the participating children gave informed written consent to use the collected data for research. The Study is registered with ClinicalTrials.gov, ID NCT01583335 on March 31^st^, 2012. The trial was retrospectively registered, because the study was initiated prior to the historical change in trial registration promotion. The authors confirm that all ongoing and related trials for this intervention are registered.

### Statistical analyses

#### Per protocol (PP) analyses

Linear regression analyses with 95% bootstrap CI were conducted to assess the effect of the intervention on PA of sports and outdoor activities combined and 4 selected types of PA categories: sports participation, outside playing time, television and computer use, and commuting choices, by comparing follow-up levels in models with adjustments for baseline level. Possible gender interaction was explored in all analyses by adding a product term to the models. Significant interactions were further examined in stratified analyses. Moreover, in order to assess whether intervention effects were modified by maternal baseline risk factors for overweight and obesity (i.e. maternal BMI, maternal educational level and maternal PA), statistical interaction was tested in all analyses by adding product terms the model, and significant interactions were examined using stratified analyses.

A total of 15 participants took part in the study together with at least one sibling. Given the small number of siblings, we chose not to perform cluster analyses. However, sensitivity analyses were conducted where all siblings were excluded. Also, as sensitivity analysis, we compared the completers of the intervention to the non-completers based on the inclusion criteria of the intervention (birthweight, maternal pre-pregnancy BMI, and maternal education level). Finally, to quantify the development in PAL over time, we conducted analyses looking at changes in outcome between baseline ad follow-up adjusted for baseline level of outcome.

#### Modified intention to treat analyses (ITT)

To assess whether selected dropout may have biased the results, all children that dropped out between baseline and follow-up were included in the analyses according to the intention-to-treat (ITT) principle [29]. Multiple imputations were used to impute values for the ITT analyses. In multiple imputations, m > 1 complete data sets are first generated. In each set of data, the missing data are substituted by imputed values that are constructed by predictive distributions for each of the missing values. Each of the completed data sets were then analyzed using standard methods, and the results from the analyses were then pooled in order to create a single set of estimates that comprised the variability associated with the missing data. For the present study, a total of *m* = 10 imputations were made (based on information on intervention group, municipality, BMI z-score, gender, age, maternal educational level, maternal BMI, maternal PAL, distance from day care and baseline measure of outcomes) using chained equations as implemented in Stata through the commands ice and mim [30]. Data were imputed for the intervention and the control group. The number of missing values ranged from 0 (0%) for group, gender, municipality and age to 235 (43.3%) for the paternal education variable. As in the PP analyses, standard linear regression analyses were used to examine differences between the intervention group and the control group using the imputed datasets.

All statistical tests were two-sided and P-values <0.05 were considered statistically significant. Analyses were performed using the statistical software package Stata 12 (StataCorp LP, College Station, Texas, USA; www.stata.com).

## Results

Information on one or more of the included PA outcomes at baseline and follow-up was available in a total of 127 children in the intervention group and 180 children in the control group.

The baseline characteristics of these children are presented in Table 2. While birthweight and maternal pre-pregnancy BMI were similar among children who had complete data at both baseline and follow-up compared to non-completers, a higher maternal educational level was found in the “completer group” (S1 Table).

**Table 2:** Baseline characteristics of the included participants stratified by intervention status. Results presented as median (5, 95 percentiles) unless otherwise stated.

		Intervention		Control	
	n	Median (5, 95 percentiles)	n	Median (5, 95 percentiles)	P
**Gender (% girls)**	127	44	180	41	0.60
**Age (y)**	127	4.0 (2.5, 5.5)	180	4.1 (2.4, 5.7)	0.96
**Sports activities (min/week)**	120	0 (0, 188)	176	0 (0, 240)	0.55
**Outdoor playing activities (min/week)**	120	233 (25, 958)	166	208 (30, 990)	0.82
**Sports and outdoor activities combined (min/week)**	127	260 (20, 990)	178	240 (30, 1020)	0.74
**Television and computer use (min/week)**	125	181 (1, 661)	175	181 1(1, 661)	0.24
**Active transport (frequency/week)**	74	5 (1, 10)	106	5(1, 10)	0.33
**Passive transport (frequency/week)**	115	9 (2, 10)	150	9 (2, 10)	0.62
**BMI z-score (SD)**	127	0.10 (-1.19, 1.19)	180	0.27 (-1.04, 1.24)	0.21
**Maternal BMI (kg/m**^**2**^**)**	125	26.4 (20.1, 42.2)	170	25.5 (20.4, 35.5)	0.23
**Maternal education (% high)**	127	76	180	80	0.36
**Maternal PAL (% high)**	120	62	178	62	0.98

In both the PP and the modified ITT analysis, the intervention group had obtained a higher total time spent on sports and outdoor activities combined per week after the 15 months intervention period, but only the ITT analysis reached statistical significance (ITT analyses: 400 min/week; 95% confidence interval (CI): 341, 459) compared to the control group (ITT analyses: 321 min/week; 95% CI: 277, 366; p = 0.02) (Tables 3 and 4).The intervention effects in regards to the separate categories of activity, e.g. participation in sports activities, engaging in outdoor activities, television viewing and computer use and means of transportation did not reach statistical significance in neither PP nor ITT analyses (Tables 3 and 4). In essence, the change analyses revealed similar results (S2 Table). There were, furthermore, no interactions with gender, age, maternal BMI, maternal educational level or maternal PAL for sports activities, outdoor activities, television viewing or computer use (Table 3). However, for active transport, there was a significant interaction with maternal education (p = 0.04), suggesting that the intervention was less beneficial regarding promotion of active transport among children of mothers with a low level of education. For passive transport, there was a significant interaction with maternal PAL (p = 0.02) (Table 3), suggesting a less beneficial effect of the intervention among children of mothers with a low PAL. The results were essentially similar in the sensitivity analyses that excluded children who participated along with at least one sibling (n = 15) (S3 Table).

**Table 3:** The effect of the Healthy Start intervention on physical activity categories. Results are presented as mean and 95% CI.

		Intervention	Control						
	N	Mean	Mean	P	P interaction	P interaction	P interaction	P interaction	P interaction
		(95% CI)	(95% CI)		(sex)	(age)	(BMI [mom])	(educ. [mom])	(PAL [mom])
**Sports and outdoor activities combined (min/week)**	304	400 (343, 457)	325 (280, 369)	0.05	0.49	0.50	0.90	0.60	0.51
**Sports activities (min/week)**	289	93 (74, 112)	73 (61, 86)	0.05	0.54	0.75	0.05	0.53	0.57
**Outdoor playing activities (min/week)**	278	318 (252, 385)	266 (224, 308)	0.20	0.56	0.21	0.51	0.89	0.34
**Television and computer use (min/week)**	299	304 (258, 350)	325 (288, 362)	0.50	0.84	0.25	0.14	0.95	0.21
**Active transport (frequency/week)**	148	6.2 (5.7, 6.8)	6.7 (6.1, 7.3)	0.33	0.43	0.19	0.32	**0.04**	0.09
**Passive transport (frequency/week)**	230	7.6 (7.2, 8.0)	7.8 (7.3, 8.3)	0.56	0.68	0.54	0.41	0.77	**0.02**

Linear regression adjusted for baseline measure of outcomes

**Table 4:** Intention to treat analysis of the effect of the Healthy Start intervention on physical activity categories. Results are presented as mean and 95% CI.

		Intervention	Control	
	n	Mean	Mean	P
		(95% CI)	(95% CI)	
Sports and outdoor activities combined **(min/week)**	543	400 (341, 459)	321 (277, 366)	0.02
**Sports activities (min/week)**	543	91 (68, 113)	73 (56, 90)	0.20
**Outdoor playing activities (min/week)**	543	316 (264, 368)	265 (209, 321)	0.19
**Television and computer use (min/week)**	543	311 (274, 349)	326 (287, 365)	0.62
**Active transport (frequency /week)**	543	5.4 (4.5, 6.4)	6.1 (5.6, 6.6)	0.19
**Passive transport (frequency /week)**	543	7.3 (6.8, 7.9)	7.3 (6.9, 7.7)	1.00

Multiple imputations were used to predict missing data.

Predicted means were calculated using linear regression adjusted for baseline measure of outcomes.

## Discussion

In this randomised family-based primary intervention among 2–6 years old normal weight Danish children who were biologically or socially predisposed to future overweight and obesity, we found that normal weight children in the intervention group obtained a higher level of sports and outdoor activities combined after the 15 months of intervention, compared to the control group. When examining time used on the individual types of activities, e.g. sports activities, outdoor playing activities, screen time and frequency of active transport separately, the change during the intervention in the estimates were all pointing in the same direction, favoring a higher activity level in the intervention group, however without being statistically significant, potentially due to lack of statistical power.

At present, no official recommendations for PA for children younger than 5 years exist in Denmark, but the current Danish national recommendations for the PAL among children older than 5 years are among other things to engage in a minimum of 60 minutes of moderate to vigorous PA per day [24], which is equivalent to 420 minutes per week. At follow-up, the intervention group was only slightly below this recommendation, with an estimated adjusted mean of sports and outdoor activities combined of 400 minutes per week (≈ 95%) versus the 325 minutes per week (≈ 76%) in the control group (ITT p = 0.02). These estimates are based on leisure time only, while the remaining activity performed in day care during week days could not be taken into account, since this information was not obtained in the Healthy Start study. However, a Danish report from SFI—The Danish National Centre for Social Research from 2014 showed that 65% of the children spend 6 to 8 hours/day in day care, while 22% spend less than 6 hours/day and 13% more than 8 hours/day in daycare [28]. If we assume that the randomization was successful, and the distribution of other variables would suggest that it was, the distribution of hours spent in day care would be expected to be similar in the intervention and control groups. Furthermore, the hours spent in day care are likely to influence how parents answer the questionnaire, but the potential misclassification error associated with this, is expected to be non-differential in the two groups. This could have led to attenuation of our results and hence made it harder to observe real effects of the intervention.

Although we cannot rule out compensation approaches at leisure time to the activity spend in day care, or whether parents to intervention children may in particular have over-reported moderate to vigorous activities or under-reported sedentary activities at the end of the interventions compared to parents of control children, as has been reported before [31], the intervention effect relying on self-report should be interpreted with cautious. On the other hand, all sub-categories of the PA pointed to a more favorable activity level among the children in the intervention group, also for the categories not related to day care participation, suggesting the observed differences between the intervention and control group were real.

Several obesity prevention intervention among young children (0–5 years) that examined changes in activity, found a difference between the intervention and control group in regards to reducing television viewing and computer use [32–35]. Moreover, a few studies found differences between the intervention and control groups in respect to changes in total PAL measured with accelerometers [33–35]. Although, one study among young children reported that intervention children had a higher performance in movement skills tests than children in the control group at six-month follow-up after adjustment for baseline performance [36]. Previous research in active commuting has primarily been performed among school children, but a systematic review including 14 interventions focusing on active transportation to and from primary school found small effect sizes, only, or non-significant effect [37], which is similar to our results. On the other hand, the vast majority of the previous intervention studies were performed among groups of children of mixed weight classes, e.g. not restricted to normal weight individuals, and may therefore have been confounded by intervention effects related to weight class induced changes on PA. Comparison to previous studies may not therefore be meaningful, and the intervention results for PA effects among normal weight young children are the first to be presented.

Also, the reported lack of intervention effect shown in studies using objective methods (accelerometer or double labelled water), compared observed effects when using subjective methods (questionnaire), may be explained by reporting bias. Indeed, previous studies have shown that parents of young children generally tend to overestimate the PAL of their children, when using self-report [38]. Even though the C-PAQ used in our study has been validated in the age group of interest [27], and has shown to be weakly to moderately correlated to accelerometer measures [39], as well as provide reliable estimates for ranking the children’s PA at a group level [27], there are well-known concerns. On the other hand, accelerometers or doubly labeled water method do not give information on the type, categories, and frequency of habitual PA during a typical week. Generally, previous obesity prevention studies have targeted mixed groups of normal, overweight and obese children, and a recent review of results from available interventions concluded that surprisingly no previous obesity prevention studies had targeted normal weight children, only [40].

A strength to the Healthy Start primary randomized controlled intervention study is therefore the inclusion of a large sample of children who were all normal weight at baseline, selected based on having a high risk profile in relation to future overweight and obesity. Furthermore, compared to previous interventions [41], the duration of the present intervention was relatively long. Moreover, the pioneering components of the intervention, by means of attempts to reduce stress in the family and improve sleep quantity and quality in the children, in addition to the more traditional components such as improvement in diet and PA can also be considered a strength. Certainly, the randomized design is a strength, as the risk of potential confounding is eliminated. However, due to the study design, health consultants were aware of the group allocation of the children, which may have increased the risk of observer bias. A priori, we accommodated this by providing detailed manuals and guidelines on how the consultations with the families in both the intervention group and the control group were to be carried out. Moreover, we cannot exclude that selection bias may have occurred, since the well-known phenomenon that socioeconomic background influences the willingness to participate, also applied for this study, as also evidenced by the higher maternal education level among the participating children than those that dropped out. On the other hand, the results were essentially similar in PP and ITT analyses, which suggest that this bias was of minor importance. In combination, these factors influence the generalizability, so the results may not be applicable to non-obesity-risk normal weight children or children from low socioeconomic families.

From a public health perspective, our results may contribute to future interventions and policy making, as the difference of approximately 10 minutes per day between the intervention and control group may be considered to be of physiological relevance, bearing in mind the overall beneficial health effects of PA [42].

## Conclusion

Among young children who were normal weight, but at high risk of becoming overweight and obese later in life, the Healthy Start primary intervention increased the total estimated, parent-reported, time spent on sports and outdoor activities combined (proxy of moderate to vigorous PA level). No individual effects were evident for the separate elements of subcategories of PA, e.g. sports participation, outdoor playing time, screen time or commuting frequency.

## Supporting information

S1 Table Distribution of the inclusion criteria: Birth weight, maternal pre-pregnancy BMI and education among completers and non-completers according. (DOCX)

S2 Table Per protocol (PP) and intention to treat analysis (ITT) of the effect of the Healthy Start intervention on physical activity categories. Results are presented as mean change and 95% CI.
(DOCX)

S3 Table The effect of the Healthy Start intervention on physical activity categories (excluding siblings). Results are presented as mean and 95% CI.
(DOCX)

S1 CONSORT Checklist CONSORT 2010 checklist. (DOC)
