# Supplementary material for: Effects of the Healthy Start randomized intervention trial on physical activity among normal weight preschool children predisposed to overweight and obesity
Source: PLoS One. 2017 Oct 9;12(10):e0185266. doi: 10.1371/journal.pone.0185266 (PMC5633144; doi:10.1371/journal.pone.0185266)
Supplement: S1 Table — (DOCX) [file pone.0185266.s001.docx]

| **S1 file:** Distribution of the inclusion criteria: birth weight, maternal pre-pregnancy BMI and education among completers and non-completers according ^1^. | | | | |
| --- | --- | --- | --- | --- |
|  | **n** | **Completers** | **Non-completers** | **p-value** |
|  |  | **%** | **%** |  |
| **Birth weight above cutoff (≥4000 g)** |  |  |  |  |
| No | 160 | 28 | 31 | 0.51 |
| Yes | 383 | 72 | 69 |  |
|  |  |  |  |  |
| **Maternal pre-pregnancy BMI above cutoff (BMI > 28 kg/m^2^)** |  |  |  |  |
| No | 234 | 55 | 56 | 0.87 |
| Yes | 186 | 45 | 44 |  |
|  |  |  |  |  |
| **Maternal education** |  |  |  |  |
| Educational level ≤ 10 years | 31 | 4 | 16 | <0.0001 |
| Educational level > 10 years | 300 | 96 | 84 |  |
| ^1^ Participants could have more than one inclusion criteria. Information on birthweight retrieved from the Danish Medical Birth Registry and the administrative birth forms was complete for all participants. Information on maternal pre-pregnancy BMI retrieved from the Danish Medical Birth Registry and the administrative birth was missing for 23% of the participants. Information on maternal educational level was only available from one municipality, and thus was missing for 61% of the participants. | | | | |
