# Supplementary material for: Effects of the Healthy Start randomized intervention trial on physical activity among normal weight preschool children predisposed to overweight and obesity
Source: PLoS One. 2017 Oct 9;12(10):e0185266. doi: 10.1371/journal.pone.0185266 (PMC5633144; doi:10.1371/journal.pone.0185266)
Supplement: S2 Table — Results are presented as mean change and 95% CI. (DOCX) [file pone.0185266.s002.docx]

| **S2 table.** Per protocol (PP) and intention to treat analysis (ITT) of the effect of the Healthy Start intervention on physical activity categories. Results are presented as mean change and 95% CI. | | | | |
| --- | --- | --- | --- | --- |
|  |  | **Intervention** | **Control** |  |
|  | **N** | **Mean** | **Mean** | **P** |
|  |  | **(95% CI)** | **(95% CI)** |  |
| **Sports and outdoor activities combined (min/week)** |  |  |  |  |
| **PP** | 304 | 36 (-21, 93) | -39 (-84, 5) | 0.05 |
| **ITT** | 543 | 47 (-18, 113) | -24 (-73, 25) | 0.07 |
| **Sports activities (min/week)** |  |  |  |  |
| **PP** | 289 | 45 (25, 64) | 26 (13, 38) | 0.05 |
| **ITT** | 543 | 47 (24, 71) | 26 (8, 44) | 0.14 |
| **Outdoor playing activities (min/week)** |  |  |  |  |
| PP | 278 | -19 (-86, 47) | -72 (-114, -30) | 0.20 |
| ITT | 543 | -17 (-72, 37) | -78 (-137, -19) | 0.14 |
| **Television and computer use (min/week)** |  |  |  |  |
| PP | 299 | 78 (32, 124) | 99 (62, 136) | 0.50 |
| ITT | 543 | 84 (46, 121) | 105 (67, 143) | 0.42 |
| **Active transport (frequency/week)** |  |  |  |  |
| PP | 148 | 0.4 (-0.2, 0.9) | 0.9 (0.2, 1.5) | 0.33 |
| ITT | 543 | 0.8 (-0.4, 2.0) | 1.1 (0.4, 1.7) | 0.69 |
| **Passive transport (frequency/week)** |  |  |  |  |
| PP | 230 | -0.4 (-0.9, -0.0) | -0.3 (-0.8, 0.2) | 0.56 |
| ITT | 543 | -0.5 (-1.0, 0.1) | -0.3 (-0.7, 0.2) | 0.58 |
| Linear regression adjusted for baseline measure of outcomes.  Multiple imputations were used to predict missing data. | | | | |
