# Supplementary material for: Effects of the Healthy Start randomized intervention trial on physical activity among normal weight preschool children predisposed to overweight and obesity
Source: PLoS One. 2017 Oct 9;12(10):e0185266. doi: 10.1371/journal.pone.0185266 (PMC5633144; doi:10.1371/journal.pone.0185266)
Supplement: S3 Table — Results are presented as mean and 95% CI. (DOCX) [file pone.0185266.s003.docx]

| **S3 table**: The effect of the Healthy Start intervention on physical activity categories (siblings excluded). Results are presented as mean and 95% CI. | | | | |
| --- | --- | --- | --- | --- |
|  |  | **Intervention** | **Control** |  |
|  | **N** | **Mean** | **Mean** | **P** |
|  |  | **(95% CI)** | **(95% CI)** |  |
| Sports and outdoor activities combined **(min/week)** | 289 | 406 (330, 482) | 333 (298, 368) | 0.06 |
| **Sports activities (min/week)** | 274 | 96 (74, 118) | 73 (57, 89) | 0.09 |
| **Outdoor playing activities (min/week)** | 264 | 323 (255, 390) | 277 (232, 322) | 0.29 |
| **Television and computer use (min/week)** | 284 | 310 (272, 348) | 323 (279, 367) | 0.64 |
| **Active transport (frequency/week)** | 144 | 6.3 (5.7, 6.9) | 6.7 (6.2, 7.2) | 0.34 |
| **Passive transport (frequency/week)** | 218 | 7.5 (7.2, 7.9) | 7.7 (7.2, 8.2) | 0.60 |
| Linear regression adjusted for baseline measure of outcomes | | | | |
